# Should synovectomy be performed in primary total knee arthroplasty for osteoarthritis? A meta-analysis of randomized controlled trials

**DOI:** 10.1186/s13018-019-1332-5

**Published:** 2019-08-29

**Authors:** Pei Liu, Feifan Lu, Jialiang Chen, Ziqi Xia, Huachen Yu, Qidong Zhang, Weiguo Wang, Wanshou Guo

**Affiliations:** 1 Beijing University of Chinese Medicine, Yinghuadong Road, Chaoyang District, Beijing, China; 2 China-Japan Friendship School of Clinical Medicine, Peking University, Yinghuadong Road, Chaoyang District, Beijing, China; 3 Graduate School, Peking Union Medical College, Beijing, China; 4 Department of Orthopaedic Surgery, Beijing Key Lab Immune-Mediated Inflammatory Diseases, China-Japan Friendship Hospital, No. 2, Yinghuadong Road, Chaoyang District, Beijing, 100029 China

**Keywords:** Synovectomy, Total knee arthroplasty, Meta-analysis

## Abstract

**Background:**

During primary total knee arthroplasty (TKA), synovectomy as a part of the procedure has been recommended to relieve pain and inflammation of the synovium, but there is a controversy about it due to increased bleeding. In this meta-analysis, the aim is to answer whether synovectomy should be performed routinely during TKA for symptomatic knee osteoarthritis (KOA).

**Methods:**

Relevant randomized controlled trials (RCTs) on synovectomy were retrieved through database searches of PubMed, Embase, Web of Science, and Cochrane Library up to February 2019. Studies that compared postoperative pain, clinical Knee Society Score (KSS), functional KSS, range of motion (ROM), drainage, pre- and postoperative hemoglobin difference, transfusion rate, operative time, and/or complications were included in the meta-analysis. Review Manager 5.3.0 was used for meta-analysis.

**Results:**

We included 5 RCTs with 542 knees. Pooled results indicated that the synovectomy group was associated with more blood loss via drainage (WMD = − 99.41, 95% CI − 153.75 to − 45.08, *P* = 0.0003) and pre- and postoperative hemoglobin difference (WMD = − 0.93, 95% CI − 1.33 to − 0.5, *P* < 0.00001), compared with the non-synovectomy group. No statistically significant differences were demonstrated between both groups in postoperative pain, clinical KSS, functional KSS, ROM, transfusion rate, or complications (*P* > 0.05).

**Conclusions:**

The current evidence demonstrates that performing synovectomy in primary TKA for symptomatic KOA does not have any clinical benefit. It increases postsurgical blood loss. Surgeons routinely undertaking synovectomy should deliberate whether this is clinically indicated and consider limiting resection, if possible.

## Introduction

Synovium is indispensable for the apposite function of the locomotor system. It secretes the slimy synovial fluid, which lubricates the joint and nourishes the articular cartilage. Pathologically, synovitis exacerbates chondral degeneration in osteoarthritis (OA) and inflammatory joint diseases [1]. Clinical [2], radiological [3], and pathological [4] evidence of inflammatory processes in OA undermine its definition as a non-inflammatory disease. For inflammatory joint diseases (such as rheumatoid arthritis), synovectomy can effectively relieve pain and improve postoperative function [5]. Could synovectomy be equally necessary for knee osteoarthritis (KOA)? The literature currently lacks agreement on this subject.

Synovectomy is performed during primary total knee arthroplasty (TKA) for KOA mainly in the light of a surgeon’s experience and preference [6]. Orthopedic surgeons administering synovectomy claim that synovitis is the main cause of knee joint swelling and pain [7]. In addition, synovial inflammation is a risk factor for unsatisfactory results after TKA [8], because it restricts the range of motion [9], and is associated with recurrent knee hemarthrosis and impingement [10]. In contrast, opponents prefer to avoid synovectomy. They question the clinical advantages of the procedure, considering that it prolongs operative time, increases blood loss, and induces infection [11, 12].

Previous studies have compared the outcomes of KOA patients after primary TKA with versus without synovectomy [13, 14]. However, these studies had the following deficiencies. First, few studies were included, and the quality was not reliable. Second, involved outcomes were inadequate, and safety of synovectomy was not performed. Third, inclusion of new studies might alter the results of previous meta-analyses. Therefore, re-evaluation of this subject is necessary. Our new meta-analysis completely retrieved the studies, selected high-quality ones, and evaluated both the effectiveness and safety of synovectomy.

## Methods

This meta-analysis was performed according to the Preferred Reporting Items for Systematic Reviews and Meta-analyses (PRISMA) checklist. No ethical approval was required.

### Literature search

We search all articles on synovectomy for treating KOA patients in electronic databases, including PubMed, Embase, Cochrane Library, and Web of Science, up to February 2019. In addition, a manual search of bibliographies of identified articles was executed to determine potentially relevant studies. A structured search was performed using this following search string: (Synovectomy OR Synovectomies OR Synovium Resection OR Resection, Synovium OR Synovium Resections [Mesh Terms]) AND (TKA OR TKR OR total knee arthroplasty OR total knee replacement [Title/Abstract]). Our search did not cover language or publication time restrictions.

### Inclusion and exclusion criteria

Studies were selected for meta-analysis if they met the following Population, Intervention, Comparator, Outcome, and Study design (PICOS) criteria: Population—KOA patients scheduled for primary TKA; Intervention—TKA with synovectomy; Comparison—TKA without synovectomy; Outcomes—post-operative pain, clinical KSS, functional KSS, ROM, drainage, pre- and postoperative hemoglobin difference, transfusion rate, operative time, and complications; Study design—RCTs.

Studies were ruled out if any of the following existed: non-conformance to inclusion criteria, low-quality RCTs and non-RCTs, undefined sample and control sources, non-therapeutic clinical studies, non-original studies, non-full-text reports, and undefined grouping.

### Data extraction

Standard data extraction was carried out to collect the following data from included trials: author’s name, publication year, sample size, age, gender, BMI, intervention, control group, outcomes, study design, and follow-up. Relevant data were extracted independently by two authors (Feifan Lu and Ziqi Xia) after all eligible studies were identified. In studies in which data were incomplete or unclear, attempts were made to contact investigators for clarification. All data were extracted; any discrepancy was cross-checked and resolved by a third author (Weiguo Wang) to reach a final consensus.

### Quality assessment

In accordance with the Cochrane Handbook for Systematic Reviews of Interventions, two reviewers (Huachen Yu and Qidong Zhang) independently assessed the risk of bias, including the following items: random sequence generation, allocation concealment, blinding of participants, blinding of outcome assessor, incomplete outcome data, reporting bias, and other bias. Each item was measured as low, unclear, or high bias. The risk-of-bias summary and risk-of-bias graph were generated with Review Manager 5.3.0 software (Nordic Cochrane Centre, Cochrane Collaboration, Copenhagen, Denmark).

### Statistical analysis

The results of selected studies were pooled for meta-analysis when two or more results were available. Continuous data were entered as means and standard deviations, and dichotomous outcomes as the number of events. Continuous outcomes were expressed as weighted mean differences (WMD) and 95% confidence intervals (CI). Dichotomous data were stated as relative ratios (RR) and 95% CI. The level of statistical significance was established at *P* < 0.05. The *Q* and chi-square tests were used to estimate statistical heterogeneity with the values of *P* and *I*^2^. If *I*^2^ was > 50% and *P* was < 0.1, a random effects model was utilized. Otherwise, a fixed effects model was applied. Publication bias was evaluated with funnel plot and Egger’s test.

## Results

### Search results

**Fig. 1:**
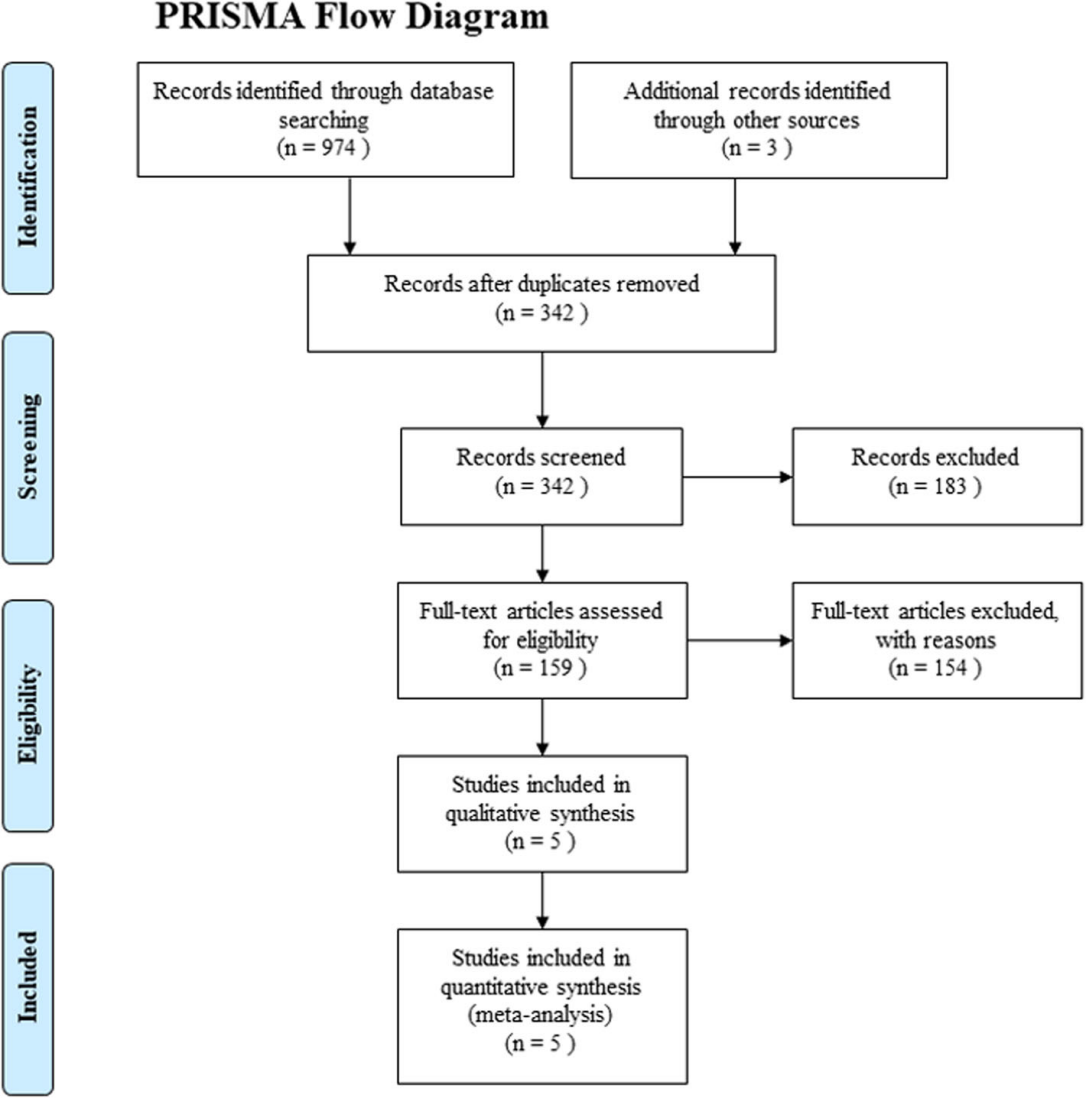
Flow diagram of study search and inclusion criteria

A total of 974 pertinent studies were identified with our search strategy; 3 additional reports were found during the manual search of references. Six hundred thirty-five duplicate studies were excluded with Endnote Software (Version X8, Thompson Reuters, CA, USA). Additional 183 studies were removed after review of the title and abstract. We excluded 154 studies after reading the full text because they did not meet the inclusion criteria. The remaining 5 studies were selected in this meta-analysis. The PRISMA flow diagram is presented in Fig. 1.

### Description of included studies and quality assessment

The including five trials [15–19] were single-centered RCTs. A total of 542 knees were involved, with an average age of 69 years, and length of follow-up from 26 weeks to 1 year. Visual analog scale (VAS) was used to evaluate postoperative pain. The most commonly adopted knee functional scores were clinical KSS, functional KSS, and ROM. Indicators of blood loss included drainage, pre- and postoperative hemoglobin difference, and transfusion rate. Two studies reported operative time and complications. The detailed information can be seen in Table 1.

**Table 1:** The general characteristics of the included studies

Study	Sample size (intervention/control)	Mean age (year)	Gender (male/female)	Mean BMI (kg/cm^2^)	Intervention	Control	Outcomes	Design	Follow-up
Bernal-Fortich et al. [16]	148 (75/73)	70	53/95	29	Synovectomy	Synovium retention	1, 6, 7, 9	RCT	1 year
Kilicarslan et al. [19]	100 (50/50)	68	30/70	NS	Synovectomy	Synovium retention	1, 3, 4, 5, 8	RCT	1 year
Rankin et al. [15]	40 (20/20)	68	29/11	28	Synovectomy	Synovium retention	4, 6, 7, 9	RCT	1 year
Tanavalee et al. [18]	67 (34/33)	70	13/54	28	Synovectomy	Synovium retention	2, 3	RCT	26 weeks
Zhaoning et al. [17]	187 (96/91)	68	48/139	27	Synovectomy	Synovium retention	1, 2, 3, 4, 5, 6, 7, 8	RCT	1 year

1, VAS; 2, clinical KSS; 3, functional KSS; 4, range of motion; 5, drainage; 6, pre- and postoperative hemoglobin difference; 7, transfusion rate; 8, operative time; 9, complication

**Fig. 2:**
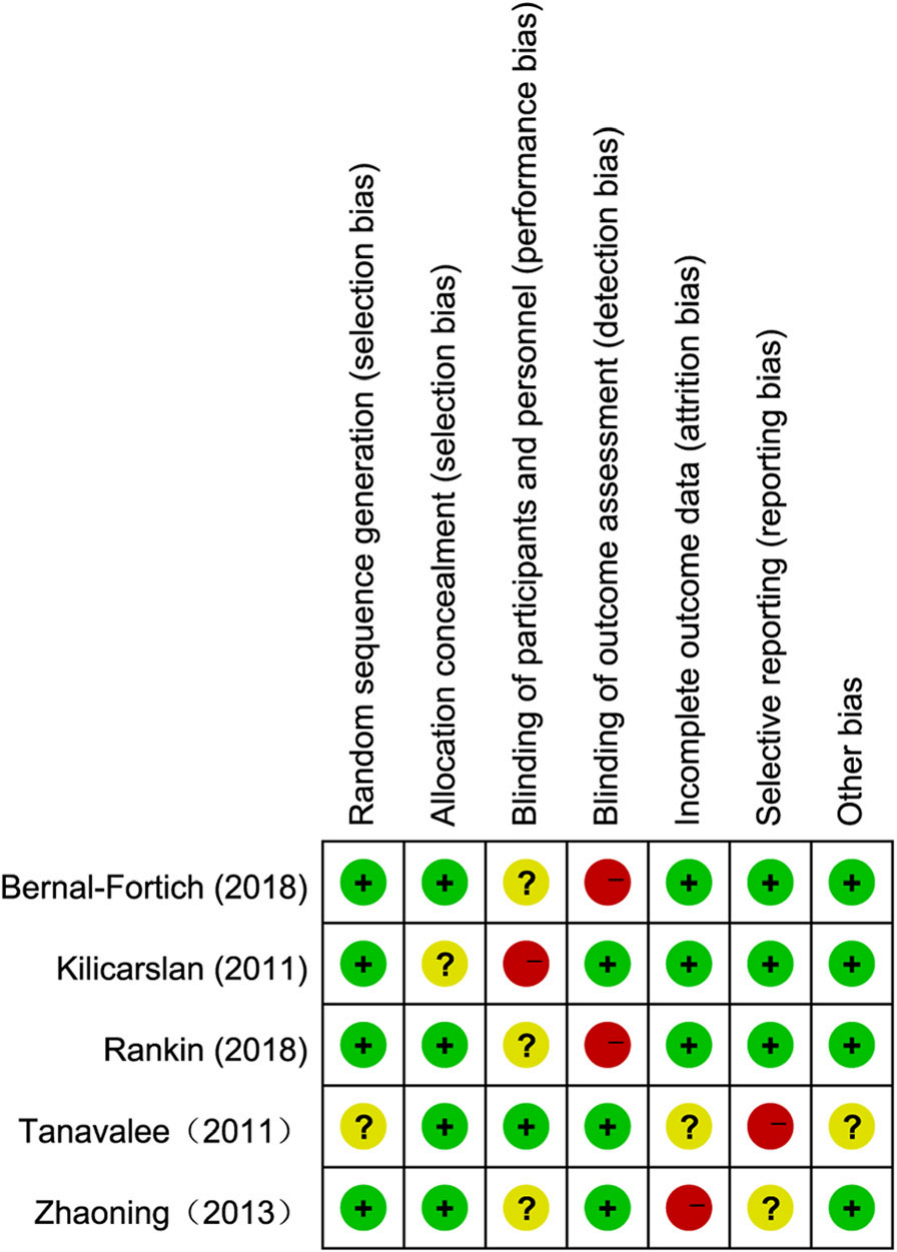
Risk-of-bias summary of included randomized controlled trials. +, no bias; −, bias; ?, bias unknown

The quality of evidence was evaluated with the Grading of Recommendations Assessment, Development, and Evaluation (GRADE) approach described in the Cochrane Handbook for Systematic Reviews of Intervention. The risk of bias varied among studies, but all contained moderate- to high-quality evidence with a low risk of bias (Fig. 2).

### Results of meta-analysis

#### Postoperative pain

**Fig. 3:**
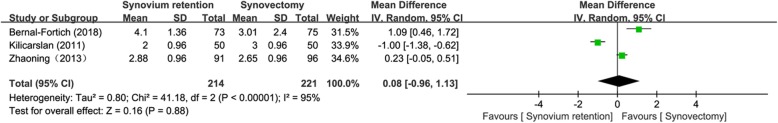
Forest plots of the included studies comparing postoperation pain

Three studies [16, 17, 19], including 435 knees, evaluated postoperative pain. Statistical heterogeneity was found in VAS (*I*^2^ = 95%, *P* < 0.00001), and a random effects model was applied. Pooled results showed no significant difference between the synovectomy group and control group in terms of postoperative pain (WMD = 0.08, 95% CI − 0.96 to 1.13, *P* = 0.88; Fig. 3**)**.

#### Postoperative function

**Fig. 4:**
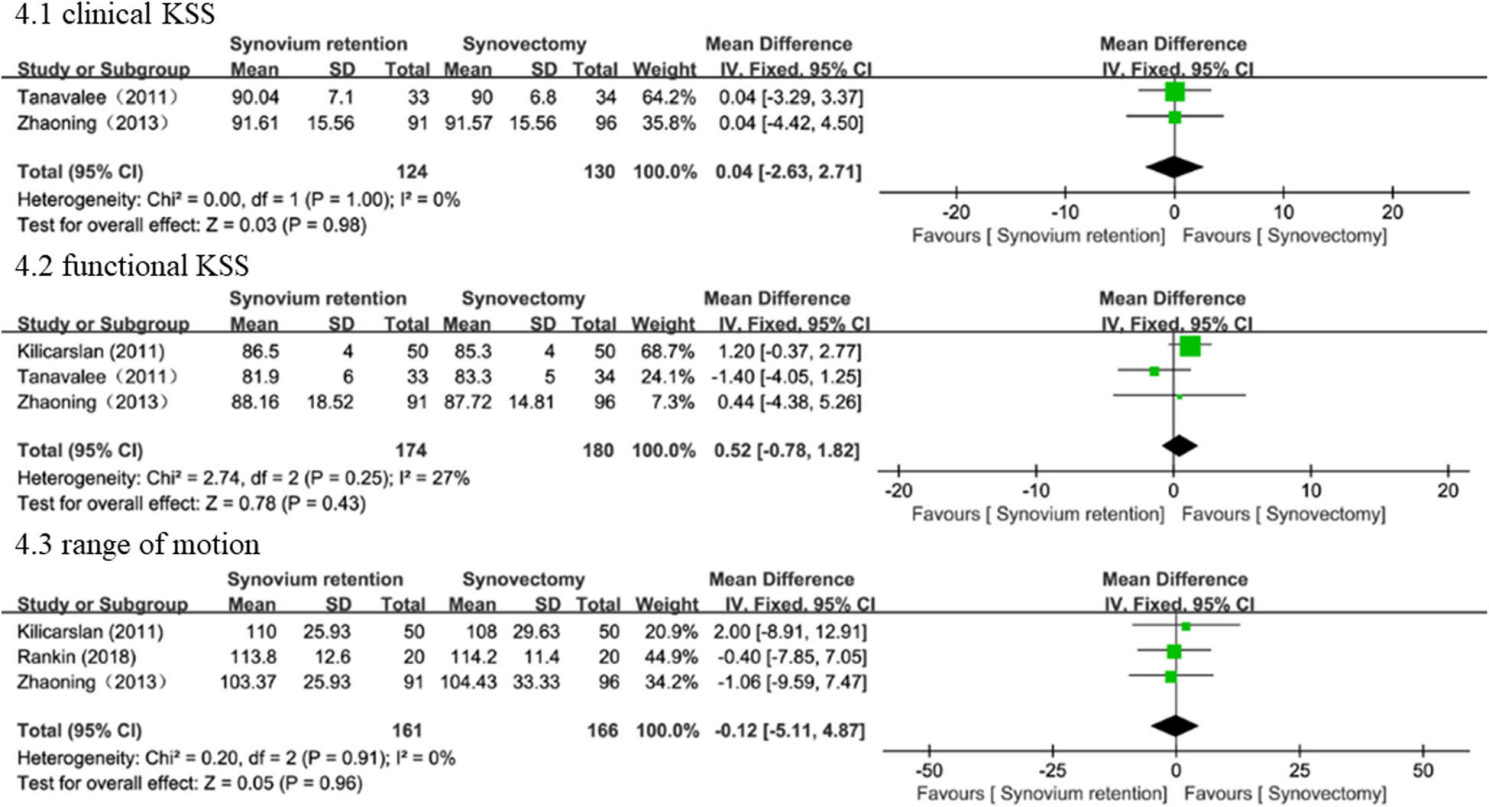
Forest plots of the included studies comparing postoperation function

We evaluated postoperative function using clinical KSS (two studies [17, 18] involving 254 knees), functional KSS (three studies [17–19] involving 354 knees), and ROM (three studies [15, 17, 19] with 327 knees). There was no significant heterogeneity between both groups, and thus, we used a fixed effects model. No significant difference was found in clinical KSS (WMD = 0.04, 95% CI − 2.63 to 2.71, *P* = 0.98), functional KSS (WMD = 0.52, 95% CI − 0.78 to 1.82, *P* = 0.43), and ROM (WMD = − 0.12, 95% CI − 5.11 to 4.87, *P* = 0.96; Fig. 4).

#### Blood loss

**Fig. 5:**
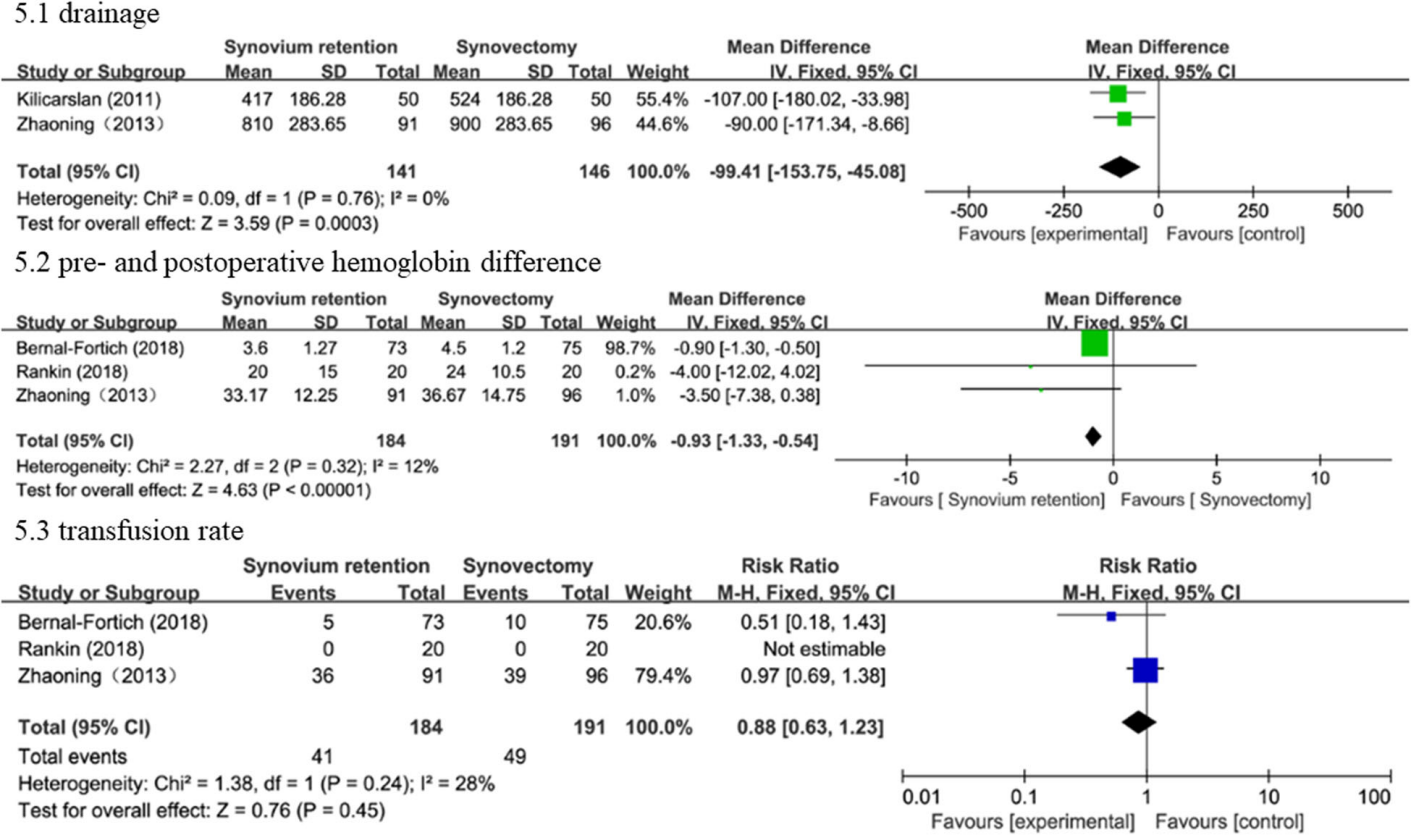
Forest plots of the included studies comparing blood loss

Blood loss was evaluated via drainage (two studies [17, 19], 287 knees), pre- and postoperative hemoglobin difference (three studies [15–17], 375 knees), and transfusion rate (three studies [15–17], 375 knees). Pooled results indicated that synovectomy was associated with greater drainage (WMD = − 99.41, 95% CI − 153.75 to − 45.08, *P* = 0.0003) and pre- and postoperative hemoglobin difference (WMD = − 0.93, 95% CI − 1.33 to − 0.5, *P* < 0.00001), but no significant difference in transfusion rate (RR = 0.88, 95% CI 0.63 to 1.23, *P* = 0.45) between the two compared groups. A fixed effects model was applied according to statistical heterogeneity. The detailed information can be viewed in Fig. 5.

#### Complications

**Fig. 6:**
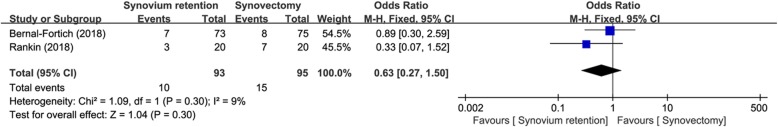
Forest plots of the included studies comparing complications

Two studies [15, 16], involving 188 knees, reported complications. A fixed effects model was utilized (*I*^2^ = 9%, *P* = 0.30). No significant difference in complications was observed between the two compared groups (RR = 0.63, 95% CI 0.27 to 1.50, *P* = 0.30; Fig. 6).

#### Publication bias and sensitivity analysis

We used Egger’s test of funnel plot to investigate the possibility of publication bias, which shows that there was no significant bias in any measured outcomes. Unfortunately, sensitivity analysis was not performed because of scant literature.

## Discussion

The main finding observed is that compared with non-synovectomy, synovectomy in primary TKA generates no clinical benefits. Our pooled data showed that synovectomy increased blood loss. In addition, we found no significant difference in regard to postoperative pain, function, and complications between the two groups.

The potential benefits of synovectomy for the management of KOA are currently debatable. In recent years, the pathogenesis of KOA is extending from a disease of cartilage to one of the “whole joint” [20]. The cartilage, bone, and synovium are each involved in pathological processes that result in advanced joint deterioration. Resection of the synovium may theoretically have beneficial effects. From a clinical standpoint, case series of arthroscopic debridement and synovectomy in the moderate to severe stages of KOA have shown some benefits [21, 22]. However, these were impermanent owing to the relentless progressive nature history of the KOA [23]. Therefore, TKA is the most common and effective selection for symptomatic end-stage KOA. Fernandez-Madrid et al. [24] suggest that synovitis might be a risk cause of pain, and it has been proposed that surgical synovectomy can relieve pain and improve function after TKA. Synovectomy has advantageous effects, but also adverse effects. Bernal-Fortich et al. [16] report that synovectomy increases the amount of bleeding after the procedure, with a difference of 9 g/l of pre- and postoperative hemoglobin. Tanavalee et al. [18] conclude that synovectomy in TKA does not shorten the duration of the inflammatory response after surgery.

Conflicting results were discovered assessing postoperative pain. The RCT of Zhaoning et al. [17] described no differences in postoperative pain at 24 h, 3 days, and 4 weeks in KOA patients with or without synovium resection (*P* > 0.05). Kilicarslan et al. [19] found that the VAS score at 3 months was lower in the synovium-retaining group. Bernal-Fortich et al. [16] reported that postoperative pain was significantly lower in the synovectomy group at 24 h and 48 h (*P* < 0.001). However, the difference was statistically significant; it was not clinically relevant. In our meta-analysis, pooled results showed no significant difference in postoperative pain between synovectomy and non-synovectomy groups. However, there was high heterogeneity among the included studies. The source of heterogeneity may be due to different analgesic methods in the perioperative period.

TKA is performed to remove damaged articular cartilage and subchondral bone and to restore alignment, which may be why the procedure is so effective for most patients. However, satisfaction analyses show that at least 20% of patients are unsatisfied with their knee outcomes [25]. We used clinical KSS, functional KSS, and ROM to evaluate the postoperative knee function. Pooled results showed no significant difference in postoperative function between both groups. Zhaoning et al. observed no difference in clinical KSS, functional KSS, and ROM between the preservation and resection group [17]. The study by Rakin et al. also found no difference in ROM (*P* = 0.602) and health score (81.00 versus 85.63) at 1 year between patients with or without synovectomy [15].

Our meta-analysis demonstrates that synovectomy can be carried out safely during TKA with no detrimental effects. The risks of synovectomy include longer operative time, extra blood loss, more blood transfusion rate, and added complications. We concluded that blood loss via drainage and pre- and postoperative hemoglobin difference was greater in the synovectomy group. Many studies agree with our findings in term of blood loss [6, 13]. An accurate synovectomy technique is vital to prevent blood loss. First, precise tissue planes are identified. Secondly, it is important to remove the subintimal and intimal layers but to leave the vascular layer intact [15]. Except for surgical technique, many risk factors such as patient’s gender, prosthesis type, femoral plug usage, additional medical comorbidities, and thromboembolism prophylaxis influence blood loss [26, 27]. Conflicting evidence was described evaluating operative time. The study by Zhaoning et al. found that the mean operative time was longer in the synovectomy group (1.50 h (1.34 to 1.75) vs 1.41 h (1.21 to 1.79), *P* = 0.006) [17]. Kilicarslan et al. observed no differences between the sides in mean operative time [19]. Synovectomy is associated with increased operative time, mainly owing to the added time of removal of the synovium and hemostasis. However, pooled data do not demonstrate that increased operative time and extra blood loss translate to more complications and blood transfusion rate.

Some limitations of this meta-analysis must be recognized. First, a relatively small number of studies and a limited number of sample sizes have weakened the objective evaluation. Second, partial RCTs lacked descriptions of random sequence generation, allocation concealment, and blinding methods, which decreased the robustness of the analysis. Third, as predictors of postoperative pain and function, presurgical patient factors were not evaluated [28] because of the lack of raw data. Fourth, the follow-up duration was relatively short; long-term follow-up was needed. Finally, the included studies used different treatment modalities of perioperative nursing and rehabilitation which could have affected results. This meta-analysis did not evaluate these factors; further evaluation is necessary in future studies.

## Conclusion

In conclusion, our meta-analysis has demonstrated that clinical outcomes between synovectomy and non-synovectomy are equivalent, with the exception of the increased blood loss. However, synovectomy is safe. It has no effect on postoperative pain and function, complications, and blood transfusion rate. While these findings may not mean the need for immediate clinical changes, research spurred by these outcomes may eventually lead to clinical changes, if these are valid.

## Data Availability

The datasets generated and/or analyzed during the current study are available from the corresponding author on reasonable request.

## Abbreviations

CI: Confidence intervals
GRADE: Grading of Recommendations Assessment, Development, and Evaluation
KOA: Knee osteoarthritis
KSS: Knee Society Score
OA: Osteoarthritis
PRISMA: Preferred Reporting Items for Systematic Reviews and Meta-analyses
RCT: Randomized controlled trial
ROM: Range of motion
RR: Relative ratios
TKA: Total knee arthroplasty
VAS: Visual analog scale
WMD: Weighted mean differences
